# An abdominal ectopic pregnancy following a frozen-thawed ART cycle: a case report and review of the literature

**DOI:** 10.1186/s12884-017-1294-8

**Published:** 2017-04-07

**Authors:** Atsushi Yanaihara, Shirei Ohgi, Kenichirou Motomura, Yuko Hagiwara, Tae Mogami, Keisuke Saito, Takumi Yanaihara

**Affiliations:** 1 Yanaihara Women’s Clinic, 1-26-29 Ofuna, Kamakura, Kanagawa Zip247-0056 Japan; 2 Department of Obstetrics & Gynecology, Women’s Health, Yokohama City University Hospital, 3-9 Fukuura Kanazawa-ku, Yokohama, Zip 236-0004 Japan

**Keywords:** Abdominal ectopic pregnancy, HCG, Laparoscope, IVF, Case report

## Abstract

**Background:**

Ectopic pregnancy (EP) occurs in 1% of pregnancies and is reported to be more common in in vitro fertilization/intracytoplasmic sperm injection (IVF/ICSI) pregnancies. An abdominal ectopic pregnancy (AEP) is a rare form of EP, and there are few reports of an AEP after IVF/ICSI. In this case report, a rare case of AEP after frozen-thawed cycle of ICSI is presented.

**Case presentation:**

After a frozen-thawed cycle of ICSI, the beta-human chorionic gonadotropin (HCG) level at 4 weeks 0 days of gestation was 3.4 IU/L. Subsequent dysfunctional uterine bleeding was mistaken for menstruation; however, an AEP of 9 weeks with a fetal heart beat was observed by ultrasound. After the AEP was observed by ultrasound, it was extracted laparoscopically.

**Conclusion:**

A rare case of an AEP, which developed after frozen-thawed cycle of ICSI, presented with a very low serum HCG level. Even if the HCG titer is low, follow-up HCG levels and frequent medical examinations are necessary.

## Background

Ectopic pregnancies (EP) occur in approximately 1% of all pregnancies, although traditionally the incidence is thought to be significantly higher in pregnancies resulting from in in vitro fertilization/intracytoplasmic sperm injection (IVF/ICSI) treatment [1, 2]. This has particularly been reported to be the case for women with tubal rather than non-tubal infertility [3, 4]. Multivariable logistic regression analyses have also accordingly demonstrated that the major risk factor for EP is the presence of tubal infertility, followed by an increased number of embryos transferred. On the other hand, extended embryo culture, such as blastocyst transfer, has shown to significantly reduce the risk of EP, while frozen-thawed embryo transfer (T-ET) has shown no effect on the risk of EP following IVF/ICSI [5–7].

As over 90% of EP are tubal pregnancies, abdominal ectopic pregnancies (AEP) constitute a rare form of EP, with increasing morbidity and difficulty in diagnosis [5, 8]. In this article a case of AEP is presented, which occurred following a single T-ET cycle of ICSI in a woman with non-tubal infertility. The beta-human chorionic gonadotropin (HCG) level was very low at 4 weeks 0 days of gestation, highlighting the difficulty such cases can pose for diagnosis and management.

## Case presentation

A 37-year-old primipara who had had three previous intrauterine inseminations (IUI) at another clinic was referred to our clinic for a second opinion. She had been undergoing infertility treatment for five years. Clinical information forwarded from the previous clinic included: semen analysis, within normal limits; hysterosalpingography, patent Fallopian tubes; serum hormone levels, within normal limits (Day 3, follicle-stimulating hormone 11.3 IU/L, luteinizing hormone 2.4 IU/L, prolactin 11.3 ng/ml; Day 20, estradiol 113 pg/ml, progesterone 15.6 ng/ml). The clinical findings at out clinic were: transvaginal ultrasound, uterine myoma (2.7 cm) in the muscular layer; endometrium, no irregularity; postcoital test, negative; and antisperm antibody, negative.

The patient underwent one IUI in our clinic; subsequently, she and her husband agreed to undergo IVF for infertility of unknown origin. The IVF process entailed ovarian stimulation with clomiphene plus human menopausal gonadotropin injections for two cycles [9]. Four ova were harvested, and three were fertilized via ICSI. Although semen analysis was previously reported as normal, ICSI was performed during the IVF/ICSI cycle due to a low motility (<10%) found during that cycle. In the next cycle, intrauterine T-ET of one blastocyst (5BA grade; Gardner criteria) was done with a hormone replacement cycle under ultrasound guidance with a soft-tipped catheter by a board-certified member of the Japan Society for Reproductive Medicine. The endometrial thickness was 10.5 mm, and the embryo was expelled into the uterine cavity approximately 1 cm from the uterine fundus with good visualization. A subsequent pregnancy test was negative, and the serum β-HCG was 3.4 IU/L at 4 weeks 0 days of gestation from T-ET (post-transfer 9 days). A re-examination was planned for day 3 of the menstrual cycle or one week if menses did not occur. Four days later, the patient called and informed us that menstruation had begun, but she was unable to come to the clinic because of her business schedule; therefore, we advised her to present at the clinic 12 days after menstruation began. At that time (post-transfer 24 days), a transvaginal ultrasound imaged a follicle-like circle with low echogenicity near the Pouch of Douglas; thus, we advised her that, based on her menstrual cycle, it was a good date for sexual intercourse. She was not able to present for her next examination for ovulation because of other commitments. Twenty-seven days after her menses began (post-transfer 40 days), she informed us that her urinary pregnancy test was positive. She presented at our clinic the following day, and a second urinary pregnancy test was also positive (post-transfer 46 day, 9 weeks 3 days of gestation).

**Fig. 1:**
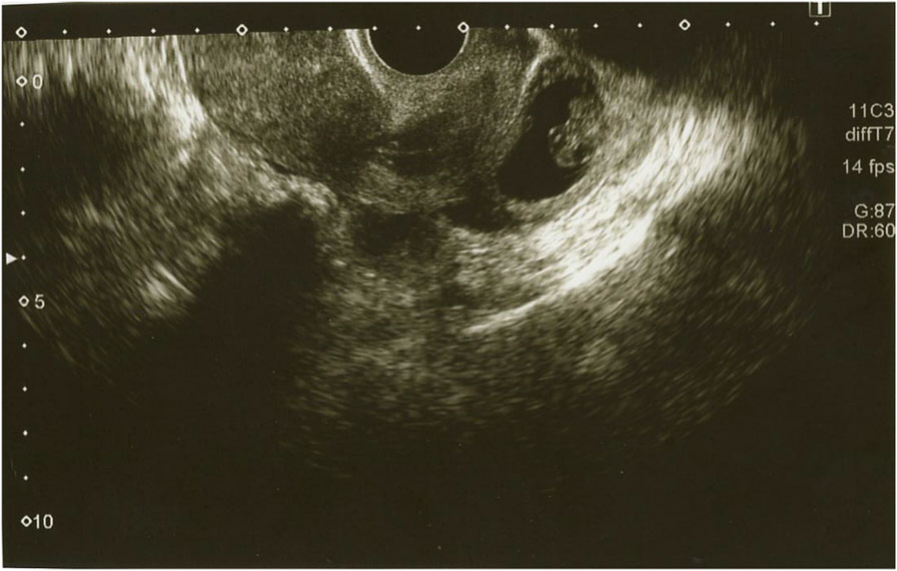
Ultrasound of gestational sac near Douglas cavum

**Fig. 2:**
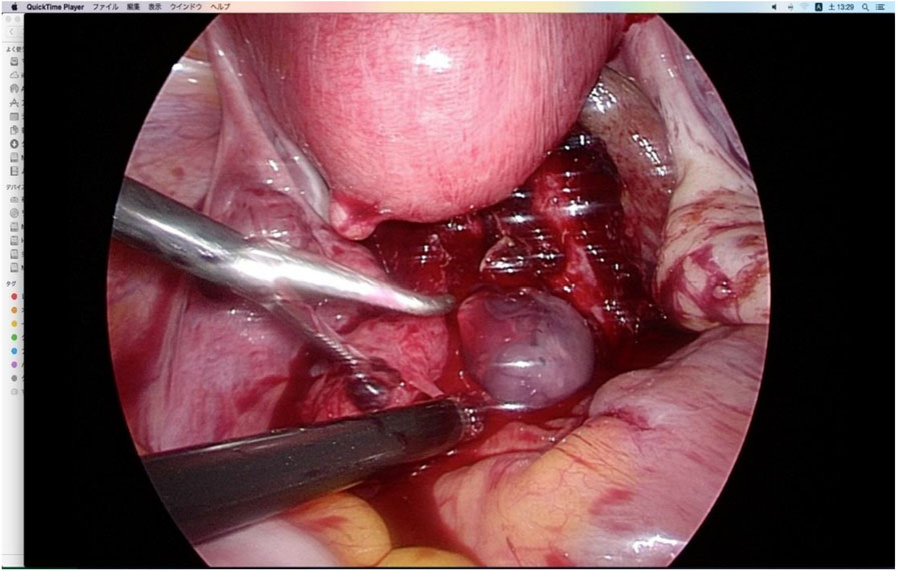
Laparoscopic view of gestational sac near Douglas cavum

Transvaginal ultrasound was performed and a gestational sac (GS) could not be located in the uterus, but there was a GS containing a fetus consistent with 8 weeks 6 days of gestation (crown rump length: 18.8 mm) near the pouch of Douglas. (Fig. 1) An EP was diagnosed, and the patient was transported to another hospital because our clinic does not have an operating room suitable for laparoscopy. The laparoscopic diagnosis was an unruptured AEP of the pouch of Douglas. (Fig. 2). The gestational tissue was removed by forceps, and electrocauterization was used for hemostasis under laparoscopic surgery. The serum β-HCG was less than 0.5 IU/L 33 days after surgery.

## Discussion and Conclusions

Recently, the diagnosis of EP has been possible before rupture because of a new technique for improving ultrasound diagnosis and improving early sensitive serum HCG measurements [10]. It is difficult to discover an EP during a spontaneous pregnancy, but, for a patient who presents regularly at a hospital during early gestation, early diagnosis is facilitated. It is sometimes still difficult to discriminate between an EP and a miscarriage during early gestation. However, the literature contains a number of reports addressing the association between EP and a frozen-thawed IVF/ICSI cycle. Many reports have suggested that frozen embryo transfer has no effect on the risk of EP [5, 11–13]. In addition, frozen-thawed day 5 blastocyst transfer is associated with a lower risk of EP than either day 3 transfer or fresh transfer [14]. A peritoneal pregnancy is rarer, and the incidence of EP has been reported to be 0.9%. It has also been reported that the mortality rate is 7.7 times higher than other types of EP [15]. It is thought that this is because intra-abdominal hemorrhage is more common in an EP than a tubal pregnancy, and the symptoms have an abrupt onset and progression. It was fortunate that an intra-abdominal pregnancy was avoided in the present case, but the risk is ever-present.

This case illustrates the following points:

1. This case was very likely to be misdiagnosed as a miscarriage because the serum β-HCG at 4 weeks 0 days from T-ET was quite low (3.4 IU/L). However, the literature contains a report of a ruptured EP with an HCG level of <10 IU/L [16]. Thus, we must keep the possibility of an EP in mind at all times, even if the HCG titer is very low.
2. The bleeding 4 days after the diagnosis of pregnancy was misdiagnosed as menstruation via a telephone conversation; however, it was actually dysfunctional uterine bleeding. This situation can often occur during a normal pregnancy.
3. A gestational sac or follicle was not detected when the patient presented at our clinic 12 days after the onset of menses. Based on the day of transfer, a yolk sac should have been observed within the echogenic area, and it should have been determined that the echogenic area was not a follicle.

It was fortunate in this case that a rupture did not occur. The pouch of Douglas might be an area where minimal friction between the EP.

There are some previous reports of EP, and many have undergone surgery or diagnostic examination laparoscopically.

**Table:** 

Author (year)	Age	Infertility	Fresh	NET	SET	Operation	Outcome
		diagnosis	/Frozen ET			/diagnosis	
Oehniger (1988) [17]	35	endometriosis	Fresh	4	2	Laparotomy	Removal of pregnancy tissue
Bassil (1991) [18]	33	male factor	Fresh	4	unknown	Laparotomy	Delivery of viable twins at 34 weeks
Ferland (1991) [19]	32	tubal factor	Fresh	3	2	Laparotomy	Removal of pregnancy tissue
Ragni (1991) [20]	32	pelvic disease	Fresh	3	2	Laparotomy	Removal of pregnancy tissue
Balmaceda (1993) [21]	33	tubal factor	Fresh	4	4	Laparoscopy	Removal of pregnancy tissue
Fisch (1995) [22]	32	tubal factor	Fresh	3	unknown	Laparotomy	Removal of pregnancy tissue
DelRosario (1996) [23]	33	tubal factor	Frozen	4	unknown	Laparoscopy	Removal of pregnancy tissue
Fisch (1996) [24]	38	tubal factor	Fresh	4	3	Laparotomy	Removal of pregnancy tissue
Moonen-Delarue (1996) [25]	23	pelvic disease	Fresh	unknown	unknown	Laparotomy	Removal of pregnancy tissue
Pisarska (1998) [26]	35	unexplained	Fresh	6	unknown	Laparoscopy	Removal of pregnancy tissue
Deshpande (1999) [27]	33	endometriosis	Fresh	2	3	Laparotomy	Removal of pregnancy tissue
Scheiber (1999) [28]	37	tubal factor	Frozen	2	3	Chemical	KCL
Dmowski (2002) [29]	34	tubal factor	Fresh	3	3	Laparotomy	Removal of pregnancy tissue
Jain (2002) [30]	29	unexplained	unknown	2	unknown	Laparotomy	Removal of pregnancy tissue
Cormio (2003) [31]	30	tubal factor	Fresh	4	3	Laparotomy	Removal of pregnancy tissue
Reid (2003) [32]	28	tubal factor	unknown	3	unkown	Laparotomy	Removal of pregnancy tissue
Kitade (2005) [33]	37	unexplained	Fresh	3	3	Laparoscopy	Removal of pregnancy tissue
Ali (2006) [15]	35	tubal factor	Fresh	1	unkown	Laparoscopy	Removal of pregnancy tissue
Apantaku (2006) [34]	33	tubal factor	Fresh	2	unkown	Laparoscopy	Removal of pregnancy tissue
Knopman (2007) [35]	37	unexplained	Fresh	2	5	Laparoscopy	Removal of pregnancy tissue
Shih (2007) [36]	33	male factor	Fresh	unkown	unkown	Laparoscopy	Removal of pregnancy tissue
Shojai (2007) [37]	35	structural	unknown	3	unkown	Laparotomy	Delivery of viable twins at 32 weeks
Iwama (2008) [38]	31	tubal factor	Fresh	3	3	Laparotomy	Removal of pregnancy tissue
Hyvarinen (2009) [39]	unknown	unknown	unknown	unknown	unknown	Laparotomy	30 weeks delivery
Zacche (2011) [40]	36	tubal factor	Fresh	2	unknown	Laparotomy	Delivery of viable twins at 30 weeks
Angelova (2015) [41]	33	male factor	Fresh	2	3	Laparoscopy	Removal of pregnancy tissue
Yoder (2016) [8]	30	male factor	Fresh	1	5	Laparoscopy	Removal of pregnancy tissue

*NET* Number of embryo(s) transferred, *SET* Stage of embryo transferred

Among the EP cases, some have been reported to continue to term [42, 43], and in EP after IVF/ICSI, there have been cases that were delivered [18].

Yoder et al. have recently reported a systematic review of 29 cases (including their case) of AEP after IVF/ICSI. According to their review, several trends of AEP were identified. The majority of cases (61%) had a history of anatomic/structural infertility with a history of tubal factor infertility (46%). This was consistent with tubal factor infertility being a known risk factor for ectopic pregnancy following IVF/ICSI. In addition, a history of tubal EP was particularly common, being reported in 37% of the abdominal ectopic cases. A history of prior tubal surgery was also particularly common (50%) among abdominal ectopic cases in their systematic review. Fresh embryo transfer was far more common in abdominal ectopic cases than frozen embryo transfer (11% of cases) [8].

The risk of EP after IVF/ICSI is increased by the number of embryos [6, 7]. As a mechanism for AEP by IVF/ICSI, aspects of the transfer that may increase the risk of EP include a large volume of transfer media, induction of abnormal uterine contractions, and the location of the embryo transfer in relation to the uterine fundus [10]. Bu et al. reported that the rate of EP was positively associated with ovarian stimulation for fresh IVF/ICSI cycles [6]. The endometrial combined thickness was also linked to an increased risk of EP [44]. Thus, it may be that EP may occur when some factors are present at the same time.

The present case was a rare occurrence, and AEP can occur even in cases where the index of suspicion would be theoretically low. The cause of AEP is unknown at present, and it is difficult to prevent. Technical improvement of IVF/ICSI may lower its incidence if IVF/ICSI is hypothesized to be one cause of EP. Further study is necessary to avoid this rare IVF/ICSI complication.

## Abbreviations

AEP: Abdominal ectopic pregnancy
EP: Ectopic pregnancy
GS: Gestational sac
HCG: Human chorionic gonadotropin
ICSI: Intracytoplasmic sperm injection
IUI: Intrauterine inseminations
IVF: Conventional in vitro fertilization
T-ET: Thawed embryo transfer
